# Crystal structure of 2-cyano-3,3-bis­(ethyl­sulfan­yl)-*N*-*o*-tolyl­acryl­amide

**DOI:** 10.1107/S2056989017005783

**Published:** 2017-04-28

**Authors:** Rasha A. Azzam, Galal H. Elgemeie, Rokia Ramadan, Peter G. Jones

**Affiliations:** a Chemistry Department, Faculty of Science, Helwan University, Cairo, Egypt; b Institut für Anorganische und Analytische Chemie, Technische Universität Braunschweig, Postfach 3329, D-38023 Braunschweig, Germany

**Keywords:** crystal structure, cyano­ketene, thio­acetal

## Abstract

In the mol­ecule of the title compound, C_15_H_18_N_2_OS_2_, the central S_2_C=C(CN)C moiety is planar (r.m.s. deviation = 0.029 Å). The C=O and C—CN groups are *trans* to each other across their common C—C bond. In the crystal, one classical and two ‘weak’ hydrogen bonds combine with borderline N⋯N and S⋯S contacts to form layers parallel to (10-2). One ethyl group is disordered over two positions with relative occupancy 0.721/0.279 (7).

## Abstract 2

In the mol­ecule of the title compound, the central S_2_C=C(CN)C moiety is planar (r.m.s. deviation = 0.029 Å). The C=O and C—CN groups are *trans* to each other across their common C—C bond. In the crystal, one classical and two ‘weak’ hydrogen bonds combine with borderline N⋯N and S⋯S contacts to form layers parallel to (10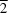).

## Chemical context   

The synthesis of ketene *S*,*S*-acetals as potential starting materials for the preparation of novel classes of heterocycles has attracted much attention (Elgemeie *et al.* 2009 ▸, 2015 ▸). As part of a research program for preparing new classes of anti­metabolites (Elgemeie *et al.* 2016 ▸, 2017*a* ▸), we have recently reported successful approaches for syntheses of pyridine, pyrimidine and mercaptopurine analogues by the reaction of cyano­ketene di­thio­acetals with active methyl­ene compounds (Elgemeie *et al.*, 2003 ▸, 2006 ▸, 2017*b* ▸). In a continuation of this research, we report here a novel cyano­ketene di­thio­acetal (1). Product (1) was prepared by the reaction of 2-cyano-*N*-(*o*-tol­yl)acetamide with carbon di­sulfide in the presence of sodium ethoxide followed by alkyl­ation with ethyl iodide. The structure of (1) was originally based on its spectroscopic data and elemental analysis (see *Experimental*). In order to establish the structure of the compound unambiguously, the crystal structure was determined.
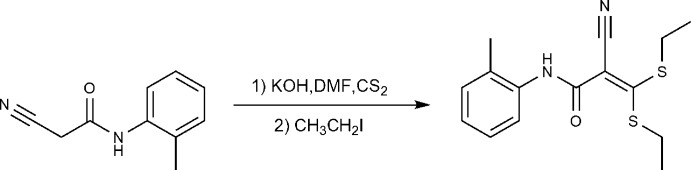


## Structural commentary   

**Figure 1:**
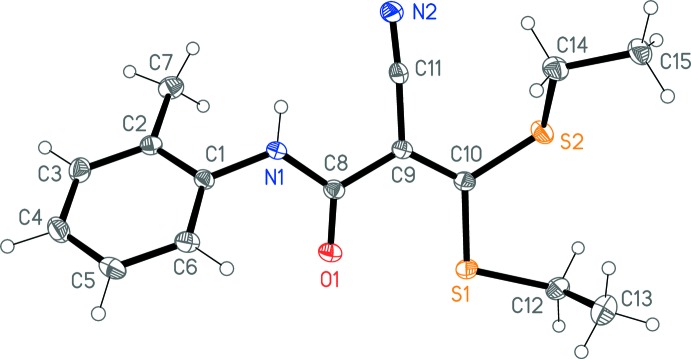
The structure of compound (1) in the crystal, with ellipsoids at the 50% probability level. Only one position of the disordered ethyl group C14/C15 is shown.

The X-ray analysis confirms the exclusive presence of the form (1) in the solid state (Fig. 1 ▸). Mol­ecular dimensions may be regarded as normal [*e.g*. C9—C10 1.3781 (16) and C9—C11 1.4290 (16) Å]. The mol­ecular backbone C1, N1, C8, C9, C10, S1, S2 is planar to within an r.m.s. deviation of 0.029 Å; O1 deviates by 0.063 (1) and C11 by 0.284 (1) Å from this plane. The aromatic ring subtends an angle of 53.30 (3)° with the same plane. The C=O and C—CN groups are *trans* to each other across the C8—C9 bond, with a torsion angle of 167.61 (11)°.

## Supra­molecular features   

**Figure 2:**
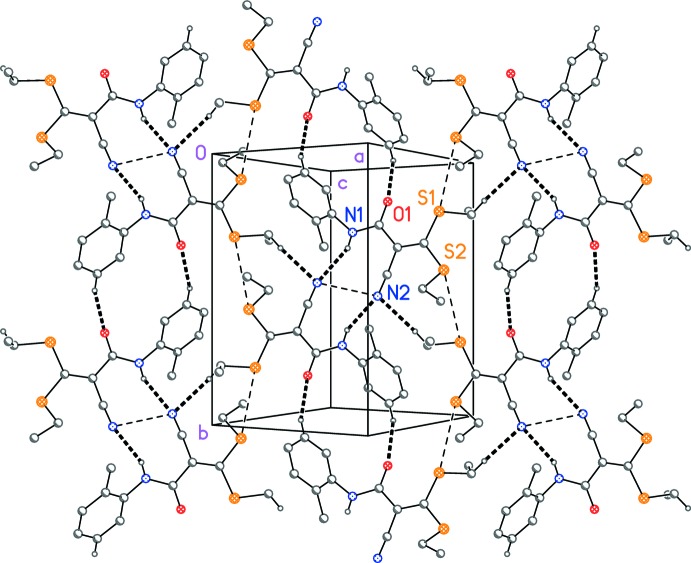
Packing diagram of compound (1) viewed perpendicular to (10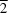). Hydrogen bonds are drawn as thick dashed bonds, with other contacts (see text) as thin dashed bonds. H atoms not involved in hydrogen bonds have been omitted for clarity.

Hydrogen bonds are given in Table 1 ▸, where the operators are also defined. The classical hydrogen bond N1—H01⋯N2^i^ connects the mol­ecules across inversion centres; associated with this inter­action, the N2 atoms of both mol­ecules are forced into a close contact of 3.061 (2) Å. Two further contacts (C—H⋯N and C—H⋯O; Table 1 ▸) may reasonably be regarded as ‘weak’ hydrogen bonds on the basis of distance and approximately linear angles at the relevant hydrogen atoms. Finally, a borderline contact S1⋯S2^ii^ of 3.7488 (4) Å is observed. All these secondary inter­actions combine to form a layer of mol­ecules parallel to (10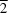) (Fig. 2 ▸).

## Database survey   

A search of the Cambridge Database (Version 1.19; Groom & Allen, 2014 ▸; Groom *et al.*, 2016 ▸) for the fragment (C—S)_2_C=C(CN)C=O gave six hits (MTBCEY, NUCFEW, SESHUT10, SESHUT11, ZAMQUZ, ZEDJEX). In all cases the C=O and C—CN groups are mutually *trans*, as in the title compound.

## Synthesis and crystallization   

2-Cyano-*N*-(*o*-tol­yl)acetamide (1 mmol) was added to a stirred solution of potassium hydroxide (2 mmol) in DMF (10 ml). After stirring for 30 min at room temperature, carbon di­sulfide (1.5 mmol) was added. The solution was left for 12 h at room temperature and then ethyl iodide (2 mmol) was added dropwise. Stirring was continued for a further 6 h. The reaction mixture was poured onto ice–water and the solid product was filtered off, dried and crystallized from ethanol to give yellow crystals, m.p. 93°C (366 K), yield 40%.

IR (KBr), 3430 (NH), 2220 (CN), 1670 (C=O) cm^−1^; ^1^H NMR (400 MHz, DMSO-*d*_6_): δ 1.25 (*t*, *J* = 8 Hz, 3H, CH_2_C*H*_3_), 1.31 (*t*, *J* = 8 Hz, 3H, CH_2_C*H*_3_), 2.51 (*s*, 3H, CH_3_), 3.03 (*q*, *J* = 6.8 Hz, 2H, C*H*_2_CH_3_), 3.12 (*q*, *J* = 6.8 Hz, 2H, C*H*_2_CH_3_), 7.15–7.36 (*m*, 4H, C_6_H_4_), 10.05 (*s*, 1H, NH), Analysis calculated for C_15_H_18_ON_2_S_2_ (306.43): C, 58.82; H, 5.88, N, 9.15, S, 20.91%; Found: C, 58.70; H, 5.65, N, 9.00, S, 20.77%.

## Refinement   

Crystal data, data collection and structure refinement details are summarized in Table 2 ▸. The ethyl group C14/15 is disordered over two positions with relative occupancy 0.721 (7)/0.279 (7). Appropriate restraints were employed to improve refinement stability, but the dimensions of disordered groups should be inter­preted with caution.

The NH hydrogen was refined freely. Methyl H atoms were refined as idealized rigid groups (C—H 0.98 Å, H—C—H 109.5°) allowed to rotate but not tip (exception: minor disorder component at C15′, set ideally staggered with AFIX 33). Other hydrogen atoms were included using a riding model starting from calculated positions, with C_arom_—H 0.95, C_methyl­ene_—H 0.99 Å, with *U*_iso_(H) = 1.5*U*_eq_(C-methyl) and 1.2*U*_eq_(C) for other H atoms.

## Supplementary Material

Crystal structure: contains datablock(s) I, global. DOI: 10.1107/S2056989017005783/hg5488sup1.cif

Structure factors: contains datablock(s) I. DOI: 10.1107/S2056989017005783/hg5488Isup2.hkl

Supporting information file. DOI: 10.1107/S2056989017005783/hg5488Isup3.cml

CCDC reference: 1544524

Additional supporting information:  crystallographic information; 3D view; checkCIF report

## Figures and Tables

**Table 1:** Hydrogen-bond geometry (Å, °)

*D*—H⋯*A*	*D*—H	H⋯*A*	*D*⋯*A*	*D*—H⋯*A*
N1—H01⋯N2^i^	0.817 (17)	2.375 (17)	3.1346 (15)	155.0 (15)
C12—H12*A*⋯N2^ii^	0.99	2.51	3.4628 (16)	160
C5—H5⋯O1^iii^	0.95	2.50	3.4110 (15)	161

Symmetry codes: (i) 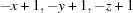; (ii) 
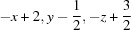
; (iii) 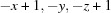.

**Table 2:** Experimental details

Crystal data	
Chemical formula	C_15_H_18_N_2_OS_2_
*M*_r_	306.43
Crystal system, space group	Monoclinic, *P*2_1_/*c*
Temperature (K)	100
*a*, *b*, *c* (Å)	9.4104 (4), 12.8350 (4), 13.0774 (5)
β (°)	104.198 (4)
*V* (Å^3^)	1531.28 (10)
*Z*	4
Radiation type	Mo *K*α
μ (mm^−1^)	0.35
Crystal size (mm)	0.35 × 0.35 × 0.30

Data collection	
Diffractometer	Oxford Diffraction Xcalibur Eos
Absorption correction	Multi-scan (*CrysAlis PRO*; Rigaku Oxford Diffraction, 2015 ▸)
*T*_min_, *T*_max_	0.986, 1.000
No. of measured, independent and observed [*I* > 2σ(*I*)] reflections	42164, 4682, 3985
*R*_int_	0.043
(sin θ/λ)_max_ (Å^−1^)	0.728

Refinement	
*R*[*F* ^2^ > 2σ(*F* ^2^)], *wR*(*F* ^2^), *S*	0.034, 0.081, 1.03
No. of reflections	4682
No. of parameters	197
No. of restraints	15
H-atom treatment	H atoms treated by a mixture of independent and constrained refinement
Δρ_max_, Δρ_min_ (e Å^−3^)	0.37, −0.22

Computer programs: *CrysAlis PRO* (Rigaku Oxford Diffraction, 2015 ▸), *SHELXS97* and *SHELXL97* (Sheldrick, 2008 ▸) and *XP* (Siemens, 1994 ▸).

## supplementary crystallographic information

### Crystal data

**Table:**

C_15_H_18_N_2_OS_2_	*F*(000) = 648
*M**_r_* = 306.43	*D*_x_ = 1.329 Mg m^−^^3^
Monoclinic, *P*2_1_/*c*	Mo *K*α radiation, λ = 0.71073 Å
Hall symbol: -P 2ybc	Cell parameters from 10087 reflections
*a* = 9.4104 (4) Å	θ = 2.7–30.4°
*b* = 12.8350 (4) Å	µ = 0.35 mm^−^^1^
*c* = 13.0774 (5) Å	*T* = 100 K
β = 104.198 (4)°	Block, pale yellow
*V* = 1531.28 (10) Å^3^	0.35 × 0.35 × 0.30 mm
*Z* = 4	

### Data collection

**Table:**

Oxford Diffraction Xcalibur Eos diffractometer	4682 independent reflections
Radiation source: fine-focus sealed X-ray tube	3985 reflections with *I* > 2σ(*I*)
Graphite monochromator	*R*_int_ = 0.043
Detector resolution: 16.1419 pixels mm^-1^	θ_max_ = 31.2°, θ_min_ = 2.2°
ω scan	*h* = −13→13
Absorption correction: multi-scan (CrysAlis PRO; Rigaku Oxford Diffraction, 2015)	*k* = −18→18
*T*_min_ = 0.986, *T*_max_ = 1.000	*l* = −18→18
42164 measured reflections	

### Refinement

**Table:**

Refinement on *F*^2^	Primary atom site location: structure-invariant direct methods
Least-squares matrix: full	Secondary atom site location: difference Fourier map
*R*[*F*^2^ > 2σ(*F*^2^)] = 0.034	Hydrogen site location: inferred from neighbouring sites
*wR*(*F*^2^) = 0.081	H atoms treated by a mixture of independent and constrained refinement
*S* = 1.03	*w* = 1/[σ^2^(*F*_o_^2^) + (0.0309*P*)^2^ + 0.7259*P*] where *P* = (*F*_o_^2^ + 2*F*_c_^2^)/3
4682 reflections	(Δ/σ)_max_ = 0.001
197 parameters	Δρ_max_ = 0.37 e Å^−^^3^
15 restraints	Δρ_min_ = −0.22 e Å^−^^3^

### Special details

**Table:**

Geometry. All esds (except the esd in the dihedral angle between two l.s. planes) are estimated using the full covariance matrix. The cell esds are taken into account individually in the estimation of esds in distances, angles and torsion angles; correlations between esds in cell parameters are only used when they are defined by crystal symmetry. An approximate (isotropic) treatment of cell esds is used for estimating esds involving l.s. planes. Least-squares planes (x,y,z in crystal coordinates) and deviations from them (* indicates atom used to define plane) 7.3265 (0.0012) x + 3.0782 (0.0029) y - 9.8498 (0.0015) z = 0.3852 (0.0018) * -0.0120 (0.0007) C1 * 0.0262 (0.0010) C8 * 0.0574 (0.0010) C9 * -0.0136 (0.0009) C10 * -0.0055 (0.0004) S1 * -0.0220 (0.0005) S2 * -0.0304 (0.0009) N1 0.0632 (0.0010) O1 0.2839 (0.0014) C11 Rms deviation of fitted atoms = 0.0287 8.1736 (0.0024) x - 6.3013 (0.0055) y - 3.6411 (0.0064) z = 1.1855 (0.0026) Angle to previous plane (with approximate esd) = 53.30 ( 0.03 ) * -0.0174 (0.0008) C1 * 0.0106 (0.0008) C2 * 0.0039 (0.0009) C3 * -0.0118 (0.0009) C4 * 0.0052 (0.0009) C5 * 0.0095 (0.0008) C6 Rms deviation of fitted atoms = 0.0107
Refinement. Refinement of F^2^ against ALL reflections. The weighted R-factor wR and goodness of fit S are based on F^2^, conventional R-factors R are based on F, with F set to zero for negative F^2^. The threshold expression of F^2^ > 2sigma(F^2^) is used only for calculating R-factors(gt) etc. and is not relevant to the choice of reflections for refinement. R-factors based on F^2^ are statistically about twice as large as those based on F, and R- factors based on ALL data will be even larger.

### Fractional atomic coordinates and isotropic or equivalent isotropic displacement parameters (Å^2^)

**Table:**

	*x*	*y*	*z*	*U*_iso_*/*U*_eq_	Occ. (<1)
C1	0.48134 (12)	0.21516 (9)	0.38738 (9)	0.0144 (2)	
C2	0.46128 (12)	0.24547 (9)	0.28216 (9)	0.0156 (2)	
C3	0.37643 (14)	0.18128 (10)	0.20464 (9)	0.0206 (2)	
H3	0.3601	0.2008	0.1326	0.025*	
C4	0.31546 (14)	0.08976 (10)	0.23047 (10)	0.0222 (2)	
H4	0.2564	0.0480	0.1765	0.027*	
C5	0.34046 (14)	0.05902 (9)	0.33513 (10)	0.0200 (2)	
H5	0.3005	−0.0045	0.3529	0.024*	
C6	0.42423 (13)	0.12172 (9)	0.41347 (9)	0.0173 (2)	
H6	0.4427	0.1008	0.4852	0.021*	
C7	0.53257 (14)	0.34242 (10)	0.25430 (10)	0.0214 (2)	
H7A	0.4999	0.4026	0.2886	0.032*	
H7B	0.5051	0.3522	0.1776	0.032*	
H7C	0.6393	0.3357	0.2784	0.032*	
C8	0.67932 (12)	0.25551 (9)	0.54338 (9)	0.0148 (2)	
C9	0.74786 (13)	0.33969 (9)	0.61750 (9)	0.0157 (2)	
C10	0.86223 (13)	0.32278 (9)	0.70449 (9)	0.0156 (2)	
C11	0.69858 (13)	0.44369 (9)	0.59035 (9)	0.0176 (2)	
S1	0.94594 (3)	0.20139 (2)	0.72800 (2)	0.01904 (8)	
C12	1.07266 (14)	0.21116 (10)	0.85705 (10)	0.0222 (2)	
H12A	1.1520	0.1594	0.8617	0.027*	
H12B	1.1179	0.2813	0.8647	0.027*	
C13	1.00032 (18)	0.19311 (12)	0.94728 (11)	0.0324 (3)	
H13A	0.9294	0.2489	0.9481	0.049*	
H13B	1.0752	0.1930	1.0142	0.049*	
H13C	0.9496	0.1258	0.9378	0.049*	
S2	0.93498 (3)	0.42489 (2)	0.79137 (2)	0.02058 (8)	
C14	0.77925 (19)	0.4973 (2)	0.81670 (17)	0.0211 (6)	0.721 (7)
H14A	0.7472	0.5518	0.7624	0.025*	0.721 (7)
H14B	0.6959	0.4496	0.8145	0.025*	0.721 (7)
C15	0.8290 (3)	0.5466 (2)	0.9252 (2)	0.0234 (5)	0.721 (7)
H15A	0.8431	0.4922	0.9793	0.035*	0.721 (7)
H15B	0.7544	0.5959	0.9358	0.035*	0.721 (7)
H15C	0.9217	0.5836	0.9306	0.035*	0.721 (7)
C14'	0.7812 (5)	0.4548 (6)	0.8430 (5)	0.0206 (13)*	0.279 (7)
H14C	0.6907	0.4573	0.7853	0.025*	0.279 (7)
H14D	0.7689	0.4007	0.8941	0.025*	0.279 (7)
C15'	0.8087 (9)	0.5593 (6)	0.8967 (7)	0.029 (2)*	0.279 (7)
H15D	0.7263	0.5771	0.9269	0.044*	0.279 (7)
H15E	0.8186	0.6125	0.8451	0.044*	0.279 (7)
H15F	0.8991	0.5563	0.9530	0.044*	0.279 (7)
N1	0.55847 (11)	0.28375 (8)	0.46806 (8)	0.0169 (2)	
H01	0.5279 (18)	0.3435 (14)	0.4655 (12)	0.026 (4)*	
N2	0.65583 (12)	0.52547 (8)	0.56292 (9)	0.0244 (2)	
O1	0.73077 (10)	0.16749 (7)	0.55038 (7)	0.01999 (18)	

### Atomic displacement parameters (Å^2^)

**Table:**

	*U*^11^	*U*^22^	*U*^33^	*U*^12^	*U*^13^	*U*^23^
C1	0.0152 (5)	0.0127 (5)	0.0153 (5)	0.0007 (4)	0.0036 (4)	−0.0020 (4)
C2	0.0157 (5)	0.0146 (5)	0.0170 (5)	0.0021 (4)	0.0048 (4)	0.0008 (4)
C3	0.0233 (6)	0.0228 (6)	0.0149 (5)	0.0019 (5)	0.0032 (4)	−0.0014 (4)
C4	0.0219 (6)	0.0191 (6)	0.0236 (6)	−0.0007 (5)	0.0018 (5)	−0.0073 (5)
C5	0.0205 (6)	0.0130 (5)	0.0272 (6)	−0.0016 (4)	0.0074 (5)	−0.0019 (4)
C6	0.0208 (6)	0.0144 (5)	0.0179 (5)	0.0010 (4)	0.0071 (4)	0.0005 (4)
C7	0.0230 (6)	0.0202 (6)	0.0221 (6)	−0.0010 (5)	0.0075 (5)	0.0048 (5)
C8	0.0166 (5)	0.0134 (5)	0.0153 (5)	−0.0007 (4)	0.0055 (4)	0.0004 (4)
C9	0.0170 (5)	0.0121 (5)	0.0172 (5)	0.0008 (4)	0.0025 (4)	−0.0005 (4)
C10	0.0150 (5)	0.0141 (5)	0.0179 (5)	0.0010 (4)	0.0046 (4)	−0.0003 (4)
C11	0.0173 (5)	0.0155 (5)	0.0175 (5)	−0.0013 (4)	−0.0007 (4)	−0.0032 (4)
S1	0.02006 (15)	0.01621 (14)	0.01943 (14)	0.00640 (11)	0.00213 (11)	−0.00021 (10)
C12	0.0184 (6)	0.0217 (6)	0.0232 (6)	0.0053 (5)	−0.0010 (4)	0.0018 (5)
C13	0.0391 (8)	0.0344 (8)	0.0218 (6)	0.0005 (6)	0.0036 (6)	0.0036 (6)
S2	0.01549 (14)	0.01860 (15)	0.02518 (15)	0.00178 (10)	0.00028 (11)	−0.00709 (11)
C14	0.0192 (9)	0.0211 (12)	0.0238 (9)	0.0043 (7)	0.0069 (6)	−0.0028 (8)
C15	0.0326 (12)	0.0214 (10)	0.0185 (11)	0.0020 (8)	0.0105 (10)	−0.0036 (9)
N1	0.0210 (5)	0.0107 (4)	0.0167 (4)	0.0021 (4)	0.0001 (4)	−0.0014 (4)
N2	0.0239 (6)	0.0151 (5)	0.0278 (5)	−0.0002 (4)	−0.0061 (4)	−0.0021 (4)
O1	0.0224 (4)	0.0132 (4)	0.0227 (4)	0.0037 (3)	0.0025 (3)	−0.0014 (3)

### Geometric parameters (Å, º)

**Table:**

C1—C6	1.3905 (16)	C3—H3	0.9500
C1—C2	1.3975 (15)	C4—H4	0.9500
C1—N1	1.4282 (14)	C5—H5	0.9500
C2—C3	1.3953 (16)	C6—H6	0.9500
C2—C7	1.5004 (16)	C7—H7A	0.9800
C3—C4	1.3850 (18)	C7—H7B	0.9800
C4—C5	1.3879 (18)	C7—H7C	0.9800
C5—C6	1.3865 (17)	C12—H12A	0.9900
C8—O1	1.2236 (14)	C12—H12B	0.9900
C8—N1	1.3585 (15)	C13—H13A	0.9800
C8—C9	1.4888 (16)	C13—H13B	0.9800
C9—C10	1.3781 (16)	C13—H13C	0.9800
C9—C11	1.4290 (16)	C14—H14A	0.9900
C10—S1	1.7390 (12)	C14—H14B	0.9900
C10—S2	1.7594 (12)	C15—H15A	0.9800
C11—N2	1.1496 (16)	C15—H15B	0.9800
S1—C12	1.8162 (13)	C15—H15C	0.9800
C12—C13	1.518 (2)	C14'—H14C	0.9900
S2—C14'	1.783 (5)	C14'—H14D	0.9900
S2—C14	1.8326 (18)	C15'—H15D	0.9800
C14—C15	1.519 (3)	C15'—H15E	0.9800
C14'—C15'	1.507 (9)	C15'—H15F	0.9800
N1—H01	0.817 (17)		
			
C6—C1—C2	120.98 (10)	C2—C7—H7A	109.5
C6—C1—N1	120.49 (10)	C2—C7—H7B	109.5
C2—C1—N1	118.50 (10)	H7A—C7—H7B	109.5
C3—C2—C1	117.77 (11)	C2—C7—H7C	109.5
C3—C2—C7	121.57 (11)	H7A—C7—H7C	109.5
C1—C2—C7	120.63 (10)	H7B—C7—H7C	109.5
C4—C3—C2	121.40 (11)	C13—C12—H12A	108.9
C3—C4—C5	120.10 (11)	S1—C12—H12A	108.9
C6—C5—C4	119.46 (11)	C13—C12—H12B	108.9
C5—C6—C1	120.21 (11)	S1—C12—H12B	108.9
O1—C8—N1	123.11 (11)	H12A—C12—H12B	107.7
O1—C8—C9	121.40 (10)	C12—C13—H13A	109.5
N1—C8—C9	115.49 (10)	C12—C13—H13B	109.5
C10—C9—C11	119.50 (10)	H13A—C13—H13B	109.5
C10—C9—C8	123.30 (10)	C12—C13—H13C	109.5
C11—C9—C8	116.99 (10)	H13A—C13—H13C	109.5
C9—C10—S1	121.02 (9)	H13B—C13—H13C	109.5
C9—C10—S2	121.14 (9)	C15—C14—H14A	110.2
S1—C10—S2	117.75 (7)	S2—C14—H14A	110.2
N2—C11—C9	176.23 (12)	C15—C14—H14B	110.2
C10—S1—C12	105.56 (6)	S2—C14—H14B	110.2
C13—C12—S1	113.23 (10)	H14A—C14—H14B	108.5
C10—S2—C14'	100.46 (17)	C15'—C14'—H14C	110.1
C10—S2—C14	107.01 (7)	S2—C14'—H14C	110.1
C15—C14—S2	107.65 (16)	C15'—C14'—H14D	110.1
C15'—C14'—S2	107.8 (5)	S2—C14'—H14D	110.1
C8—N1—C1	123.68 (10)	H14C—C14'—H14D	108.5
C4—C3—H3	119.3	C14'—C15'—H15D	109.5
C2—C3—H3	119.3	C14'—C15'—H15E	109.5
C3—C4—H4	120.0	H15D—C15'—H15E	109.5
C5—C4—H4	120.0	C14'—C15'—H15F	109.5
C6—C5—H5	120.3	H15D—C15'—H15F	109.5
C4—C5—H5	120.3	H15E—C15'—H15F	109.5
C5—C6—H6	119.9	C8—N1—H01	120.2 (11)
C1—C6—H6	119.9	C1—N1—H01	116.0 (11)
			
C6—C1—C2—C3	2.91 (17)	C11—C9—C10—S2	7.34 (16)
N1—C1—C2—C3	−175.34 (11)	C8—C9—C10—S2	−178.08 (9)
C6—C1—C2—C7	−175.44 (11)	C9—C10—S1—C12	−173.24 (10)
N1—C1—C2—C7	6.31 (16)	S2—C10—S1—C12	10.20 (9)
C1—C2—C3—C4	−0.85 (18)	C10—S1—C12—C13	83.40 (11)
C7—C2—C3—C4	177.48 (12)	C9—C10—S2—C14'	64.9 (3)
C2—C3—C4—C5	−1.27 (19)	S1—C10—S2—C14'	−118.6 (3)
C3—C4—C5—C6	1.35 (19)	C9—C10—S2—C14	44.96 (14)
C4—C5—C6—C1	0.68 (18)	S1—C10—S2—C14	−138.48 (11)
C2—C1—C6—C5	−2.86 (18)	C10—S2—C14—C15	152.32 (19)
N1—C1—C6—C5	175.35 (11)	C14'—S2—C14—C15	78.5 (5)
O1—C8—C9—C10	−7.09 (18)	C10—S2—C14'—C15'	−164.9 (5)
N1—C8—C9—C10	173.60 (11)	C14—S2—C14'—C15'	−53.9 (6)
O1—C8—C9—C11	167.61 (11)	O1—C8—N1—C1	−1.97 (18)
N1—C8—C9—C11	−11.70 (15)	C9—C8—N1—C1	177.32 (10)
C11—C9—C10—S1	−169.10 (9)	C6—C1—N1—C8	57.60 (16)
C8—C9—C10—S1	5.47 (16)	C2—C1—N1—C8	−124.15 (12)

### Hydrogen-bond geometry (Å, º)

**Table:**

*D*—H···*A*	*D*—H	H···*A*	*D*···*A*	*D*—H···*A*
N1—H01···N2^i^	0.817 (17)	2.375 (17)	3.1346 (15)	155.0 (15)
C12—H12*A*···N2^ii^	0.99	2.51	3.4628 (16)	160
C5—H5···O1^iii^	0.95	2.50	3.4110 (15)	161

Symmetry codes: (i) −*x*+1, −*y*+1, −*z*+1; (ii) −*x*+2, *y*−1/2, −*z*+3/2; (iii) −*x*+1, −*y*, −*z*+1.
